# Fanconi Anemia complementation group C protein in metabolic disorders

**DOI:** 10.18632/aging.101487

**Published:** 2018-06-21

**Authors:** Manoj Nepal, Chi Ma, Guoxiang Xie, Wei Jia, Peiwen Fei

**Affiliations:** 1 University of Hawaii Cancer Center, Honolulu, HI 96813, USA; 2 Graduate Program of Molecular Biosciences and Bioengineering, University of Hawaii, Honolulu, HI 96822, USA

**Keywords:** cancer, aging, metabolites, inflammation, diabetes, Fanconi Anemia (FA)

## Abstract

Given importance of 22-Fanconi Anemia (FA) proteins together to act in a signaling pathway in preventing deleterious clinical symptoms, e.g. severe bone marrow failure, congenital defects, an early onset of aging and cancer, studies on each FA protein become increasingly attractive. However, an unbiased and systematic investigation of cellular effects resulting from each FA protein is missing. Here, we report roles of FA complementation C group protein (FANCC) in the protection from metabolic disorders. This study was prompted by the diabetes-prone feature displayed in FANCC knockout mice, which is not typically shown in patients with FA. We found that in cells expressing FANCC at different levels, there are representative alterations in metabolites associated with aging (glycine, citrulline, ornithine, L-asparagine, L-tyrosine, L-arginine, L-glutamine, L-leucine, L-isoleucine, L-valine, L-proline and L-alanine), Diabetes Mellitus (DM) (carbon monoxide, collagens, fatty acids, D-glucose, fumaric acid, 2-oxoglutaric acid, C3), inflammation (inosine, L-arginine, L-isoleucine, L-leucine, L-lysine, L-phenylalanine, hypoxanthine, L-methionine), and cancer ( L-methionine, sphingomyelin, acetyl-L-carnitine, L-aspartic acid, L-glutamic acid, niacinamide, phospho-rylethanolamine). We also found that FANCC can act in an FA-pathway-independent manner in tumor suppression. Collectively, featured-metabolic alterations are readouts of functional mechanisms underlying reduced tumorigenicity driven by FANCC, demonstrating close links among cancer, aging, inflammation and DM.

## Introduction

Aided by new biochemical and molecular biological tools, studies in cancer cell metabolism have expanded the understanding of the mechanisms and functional consequences of tumor-associated metabolic alterations at various stages of tumorigenesis. In particular, these alterations can affect the metabolite influx through conferring an increased ability to acquire the necessary nutrients. They can also shape the manner the nutrients are preferentially assigned to metabolic pathways, which ultimately contribute to and lead to long-ranging effects on cellular fates [1]. Alterations in various metabolites also cause illnesses, such as diabetes [2] and inflammation [2], that ultimately contribute to the high incidence of cancer. During the past decade it became clear that inflammation is a key feature of obesity and type 2 diabetes [3]. Obesity is associated with an array of additional health problems, including an increased risk of insulin resistance, type 2 diabetes, fatty liver disease, atherosclerosis, degenerative disorders including dementia, airway diseases and cancers [4].

Fanconi anemia (FA), a rare human genetic disease that affects approximately 1−3 of 500000 newborns [5], is characterized by a broad spectrum of congenital abnormalities such as an early onset of aging, short stature; abnormalities of the skin, arms, head, eyes, and kidneys; bone marrow failure; a predisposition to hematological and solid malignancies. Cells derived from FA patients are featured with spontaneous and induced chromosomal breakages and very sensitive to DNA damage agents [6–8]. To date, twenty-two FA genes including FANC-A, -B, -C, -D1, -D2,-E, -F, -G, -I, -J, -L, -M, -N, -O, -P, -Q, -R, -S, -T,-U,-V and W have been identified [7–11]. These twenty-two FA genes encoded proteins are involved in a common signaling pathway (namely the FA pathway), which is activated upon DNA damage or during DNA synthesis [8,12,13]. Therefore, the absence or dysfunction of any one of these proteins can drastically reduce DNA repair efficiency or affecting DNA replication [6,8]. When this process is impaired, an accumulation of chromosomal breakage occurs, leading to genomic instability, aging, and, ultimately, cancer [14]. However, it is not clear whether defected FA genes can contribute to the onset of obesity, diabetes and/or inflammation, which are known of being closely related to cancer [3,4].

Recently, we reported that FAVL impaired FA pathway contributes to bladder cancer development at the metabolic level [15]. Those studies we did previously [16,17] hold a profound implication of functional and genetic evidences that the FA pathway performs tumor suppressor functions in cancer patient without FA, which are now extended to the exciting field of metabolomics as a result of what we recently reported [15]. In this study, we continued this new niche of metabolomics studies on FA signaling research by demonstrating how FANCC affects metabolism in a system with a defected FA signaling pathway. This was incited by the unique diabetes-prone phenotype displayed by FANCC-knockout mice [18], which is not a typical phenotype associated with FA. We found metabolites driven by FANCC are associated not only with cancer or aging but also clearly with inflammation and diabetes, which are certainly part of mechanisms for reduced tumorigenicity initiated by FANCC. Our study provides the first snapshot of all cellular processes initiated from FANCC, suggesting distinct connections among inflammation, DM, aging and cancer.

## RESULTS

### Establishment of cells expressing different levels of FANCC, which can modulate tumorigenicity

**Figure 1:**
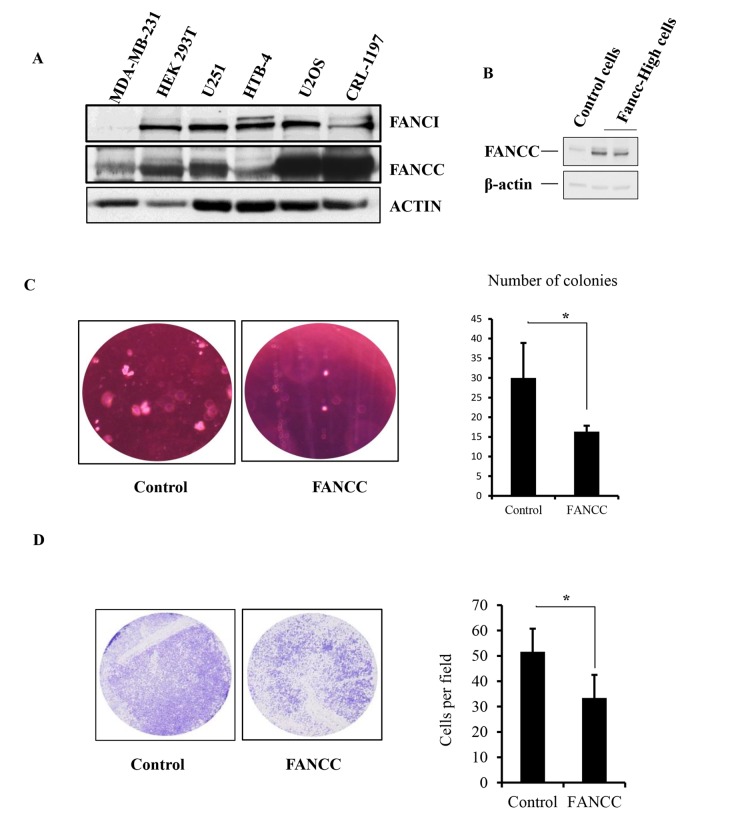
**Generation of a study system with FA-signaling-defective cells.** (**A**) MDA-MB-231 cells do not have the endogenous expression of FANCI, but have a relatively low level of FANCC expression. We examined the expression of FANCI and FANCC by western blotting in MDA-MB-231, HEK 293T, U2OS, CRL-1197, HTB-4, and U251 cells, among which the level of FANCI protein expression is undetectable in MDA-MB-231cells. However, the level of FANCC expression was low compared to the other cells detected. (**B**) Ectopic expression of FANCC in MDA-MB-231 cells. Cells were transfected with empty vectors for control or plasmids carrying FANCC cDNA. The level of FANCC was detected accordingly by western blotting in pool-selected two groups of cells. These derivatives of MDA-MB-231 express FANCC at a low or high level (namely FANCC-low or -high cells). (**C**) Number of colonies derived from FANCC-high cells is reduced compared to FANCC-low cells. Colony formation of FANCC-high or low cells was determined by the soft agar assay. The number of colonies was counted and showed significantly different between two groups (*p*<0.001). (**D**) FANCC-high cells have a reduced capacity for migration. Both cell images and numbers of migrated cells showed that FANCC can decrease the capacity of MDA-MB231 cell migration. The number of migrated cells was counted and showed significantly different between two groups (*p*<0.001).

Metabolomics study has become a powerful aspect of research to further our understanding of the basic, biological mechanisms underlying obesity, diabetes, stroke or cancer [19]. In the recognition of the diabetes-prone phenotype in FANCC knockout mice [18], that is not a typical clinical complication shown in FA, we decided to metabolically probe the function of FANCC to broaden the knowledge learned from FA. To avoid the influence of the FA signaling pathway and to systematically study the unique chemical fingerprints driven by FANCC only, we chose cells that carry an impaired FA pathway and also are easily to be built into the corresponding derivative cells to express FANCC at different levels. As shown in Figure 1A and Figure S1A, B, MDA-MB-231 and its derivative cells were validated with an undetectable level of FANCI expression, agreed with the absence of normal chromosome 15 (ATCC) where FANCI locus is located. Therefore, MDA-MB-231 cells essentially carry a non-functioning FA pathway in regards to the fact that FANCI is a partner of FANCD2 to work at the center of the FA signaling pathway [7,20]. FANCC-high or low expression cells were subsequently established from MDA-MB-231cell line (Figure 1B). Interestingly, the expression level of FANCC protein is relatively low in MDA-MB-231 cells compared to the cell lines detected (Figure 1A). This background of MDA-MB-231 cells may have also conferred the effects driven by the ectopically expressed FANCC. Indeed, those FANCC- high cells proliferated relatively slow, formed less number of colonies in soft agar, and carried a reduced migration capacity (Figure 1C, D and Figure S1A, D), suggesting that FANCC can reduce the tumorigenicity of MDA-MB-231 cells.

### FANCC-driven metabolomes

**Figure 2:**
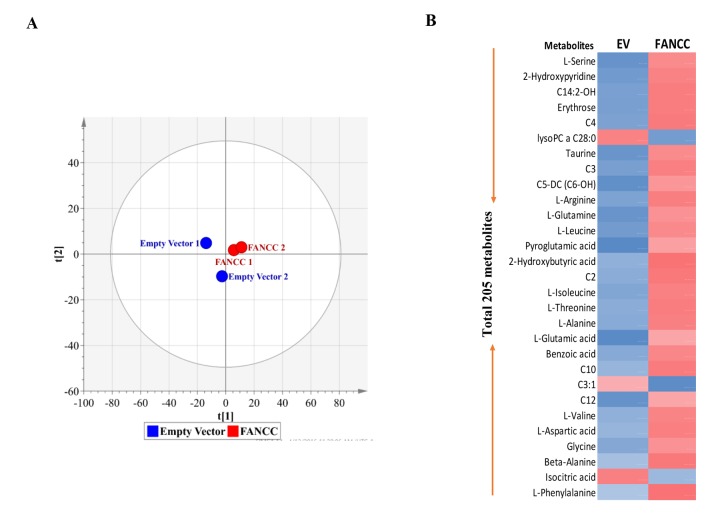
**Metabolically profiling of FANCC-high or low cells.** (**A**)OPLS-DA score plots of metabolic profiles were derived from the data of GC-TOMS, which were generated from FANCC-high or low cells. (**B**)The heat-map for the intensity variations of the total metabolites identified with GC-TOFMS and UPLC-MS/MS. Blue or red color indicates reduced or elevated levels of the metabolites respectively in FANCC-high cells compared to FANCC-low cells.

Using targeted (UPLC-MS/MS) and untargeted (GC-TOFMS) metabolomics analysis, we determined the metabolite alteration within the set of MDA-MB-231 cells expressing FANCC at different levels (Figure 1B). A total of 205 metabolites were found to be altered significantly between FANCC-high and FANCC-low cells (Table S1). Principal component analysis (PCA) showed a clear separation between empty vector control/FANCC-low and FANCC-high cell groups, providing the evidence that FANCC changes metabolomes (Figure 2A). Heat map made of 29 metabolites (randomly picked up from 205) shows a vivid difference in the metabolomes of two cell groups (Figure 2B): blue color of metabolites indicates reduced metabolites in the metabolomes of cells; while red color indicates those elevated metabolites. Therefore, FANCC at least performs biologic functions that can ultimately lead to the changes of 205 metabolites, which are a better output to tell the occurred cellular processes initiated by FANCC, in an FA pathway-independent manner.

### Involvement of FANCC in multi-aspects of cellular processes

To investigate the roles of metabolite alterations triggered by FANCC in the diseases with metabolite dysfunction such as diabetes, inflammation and cancer, we performed molecular pathway and network analyses using Ingenuity Pathway Analysis (IPA). We found that inosine, hypoxanthine, pyrophosphate, L-asparagine, L-phenylalanine, acetyl-L-carnitine, beta-alanine, glutaryl carnitine, niacinamide and creatinine significantly went up in FANCC-high cells compared to FANCC-low cells. IPA revealed that tRNA (19/43), Superpathway of citrulline Metabolism (9/24), Arginine Biosynthesis IV (7/18), Asparagine Biosynthesis (5/8) and Urea cycle (6/14) were the five most significantly altered pathways in cells expressing FANCC at a higher level compared to the control cells (FANCC-low cells) (Figure S2 and Table S2). Furthermore, we found that different metabolites are involved in different types of cellular functions. Among all metabolites, 34% are involved in cellular compromise, lipid metabolism, small molecular biochemistry, 23% are found to be involved in amino acid metabolism, molecular transport. Similarly, 22% are associated with nucleic acid metabolism, 18% is in cellular movement and 3% is related to cellular growth proliferation, organism development, injury and abnormalities (Figure S3 and Table S3). These results provide information suggesting FANCC is related to different diseases by regulating different molecular and cellular functions.

### Functional pathways initiated from FANCC

To analyze the specific relations of metabolites in the cells with FANCC potentially for different diseases, we uploaded the identified 205 metabolites to the IPA for functional analysis. For the robustness of the IPA, we normalized metabolic data as described previously [21] on the basis of a total number of cells prepared for the profiling. The metabolic functions (canonical pathways, disease and Bio-functions, molecular and cellular functions, physiological system development and functions, clinical chemistry and hematology, cardiotoxicity, hepatotoxicity and nephrotoxicity) are summarized in Tables S1 and S2. The most significantly biological functions associated with FANCC were cancer (p=4.39×10 ^-02^– 5.93X10 ^-14,^ 30 metabolites), inflammatory disease (p=3.67×X10 ^-02^–3.39×10 ^-07^, 7 metabolites) gastrointestinal disease ( p=4.39×10 ^-02^– 5.93×10 ^-14^, 28 metabolites), organismal injury and abnormalities (p=4.4×10 ^-02^– 3.39×10 ^-07^, 7 metabolites) amino acid metabolism (p=3.89×10 ^-02^ – 2.43×10 ^-10^, 21 metabolites) and liver inflammation/ Hepatitis (p=3.03×10 ^-01^ – 3.67×10 ^-02^ , 3 metabolites). All of these processes relatively went down owing to the higher expression of FANCC in comparison with FANCC-low cells. The five significant biological processes were cellular growth and proliferation (30 metabolites), amino acid metabolism (21 metabolites), molecular transport (33 metabolites), Small Molecule Biochemistry (36 metabolites) and cell cycle (13 metabolites). We also determined the most elevated molecules that are inosine, hypoxanthine, pyrosphosphate, L-asparagine, L-phenylalanine, acetyl-L-carnitine, niacinamide, glutarylcarnitine, and creatinine. These metabolites are involved in the suppression of cancer, inflammation as well as diabetes.

### Altered metabolites involved in cancer, inflammation, aging and DM

**Figure 3:**
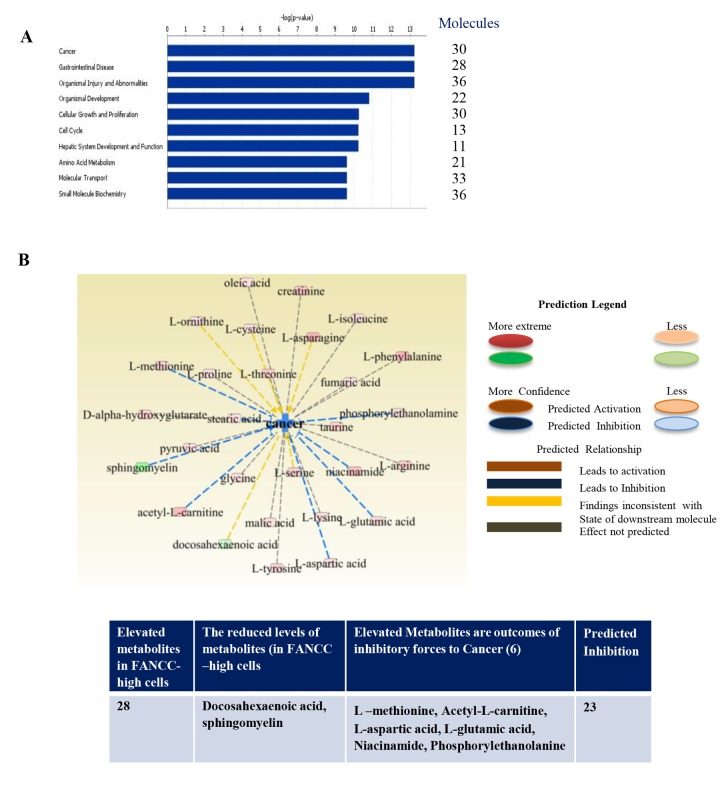
**Metabolic functions and integrative molecular modeling for different diseases.** (**A**) Top networks of metabolite markers, associated with molecular and cellular functions, were determined to be readouts of reduced cell signaling processes that are essentially to cancer and many specific disorders (gastrointestinal disease, organismal injury and abnormalities and others). (**B**) Altered metabolites were associated with cancer in FANCC-high cells in comparison with FANCC-low cells. The pink color-indicated metabolites (L-methionine, sphingomyelin, acetyl-L-carnitine, L-aspartic acid, L-glutamic acid, niacinamide and phosphorylethanolamine) were found to be elevated, which were the products of cancer inhibitory processes. Two metabolites labeled with green color were found to be lower, which were the end chemical substances of cancer promoting cellular processes. As summarized in the table shown at the bottom, 28 out of 30 metabolites were elevated in FANCC-high cells, and two were present at a lower level. Among 28 metabolites, 7 metabolites were known to be the readouts of cancer inhibitory cellular processes, and the rest of 23 were predicted to be in the inhibitory processes to cancer.

Continuing our metabolomics studies [19] on the roles of the FA signaling pathway in human tumorigenesis, we here focused on an individual FA protein and studied the pathway-free roles of FANCC in a unique cell system in which the FA pathway is deficient (Figure 1). Using IPA analysis we found that cells with extopically expressed FANCC carried either elevated metabolites that are outcomes of tumor inhibitory forces, or the decreased metabolties that are driven by oncogenic potentials. Overall, the cancer promotion signaling was prohibited as shown in red color in FANCC-high cells compared with FANCC-low cells. Other listed pathways were found to be similar mitigated (Figure 3A). Specifically, we found that 30 metabolites were specifically involved in cancer (Figure 3B). Among these metabolites, docosahexaenoic acid [22] and sphingomyelin [23] were shown at a reduced level in FANCC-high cells, which are indicative of the cellular processes that promote cancer development. While L –methionine [24], acetyl-L-carnitine [25], L-aspartic acid [26], L-glutamic acid [27], niacinamide [28] and phosphorylethanolanine [29] were clearly elevated, which are symbolized for cancer inhibitory processes. Therefore, FANCC, besides joining the FA pathway, can also play important pathway-independent roles in the suppression of cancer formation.

**Figure 4:**
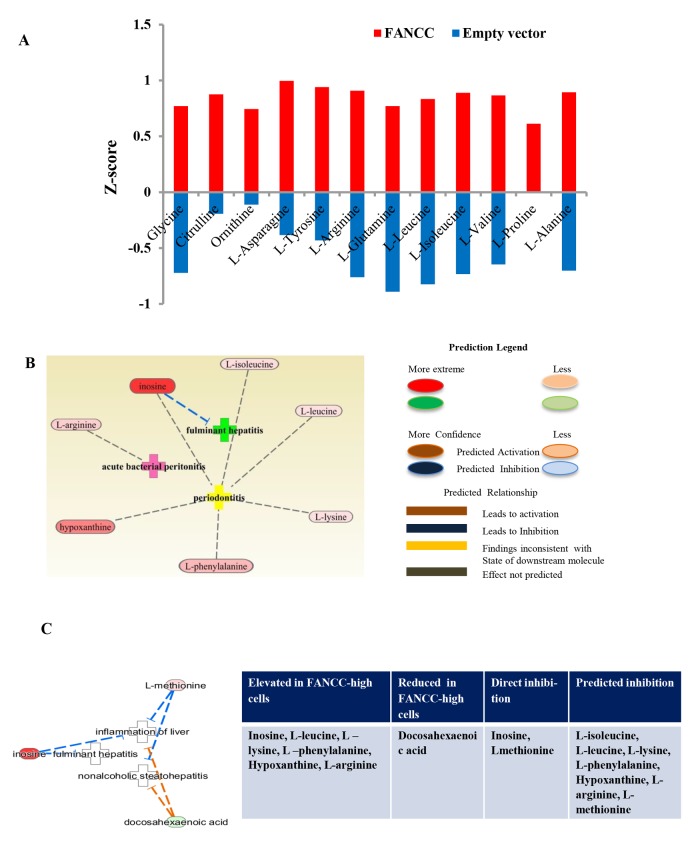
**Metabolic indication of FANCC functions in aging and inflammation.** (**A**) Aging associated metabolites were altered in FANCC-high cells. Metabolites of aging inhibitory processes were significantly elevated in FANCC-high cells. Glycine, citrulline, ornithine, L-asparagine, L-tyrosine, L-arginine, L-glutamine, L-leucine, L-isoleucine, L-valine, L-alanine and L-proline were elevated in FANCC –high cells compared to FANCC-low cells. (**B**, **C**) Metabolites involved in inflammatory diseases: The most significantly differential network for inflammatory diseases, such as periodontitis, fulminant hepatitis and inflammation of liver, was indicated by the altered metabolites in FANCC-high cells. Inosine, L-isoleucine, L-leucine, L-lysine, l-phenylalanine, hypoxanthine and L-arginine were elevated in FANCC cells, which were involved in the inflammatory-inhibition processes and etc., as listed in the table shown at the side.

**Figure 5:**
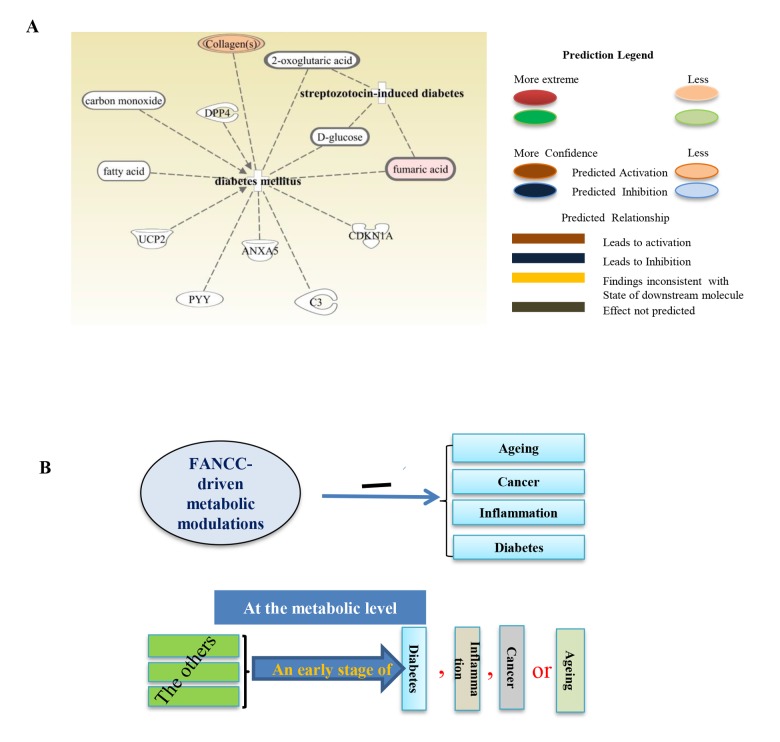
**Metabolites associated with diabetes.** (**A**) Metabolites such as collagen, 2-oxoglutaric acid, D-glucose, fumaric acid, C3, CDKN1A, ANXA5, PYY, UCP2, fatty acid and carbon monoxide were found either elevated or reduced, which are accordingly for DM-inhibitory or–promoting cellular activities (Collagens and fumaric acid were elevated). (**B**) The schematic representation of FANCC working model. FANCC-driven metabolic modulations are the common protection for humans from cancer, aging, inflammation and DM. Each of these diseases, before the occurrence of its “symptoms”, can be designated by some common metabolic alternations, appearing to be an initial stage of the subject disease. At this phase of common changes, a given “disease or symptom” may be reversible before its onset.

We next checked the distinct metabolites associated with other metabolic disorders. As shown in Figure 4A, metabolites- glycine, citrulline, ornithine, L-asparagine, L-tyrosine, L-arginine, L-glutamine, L-leucine, L-isoleucine, L-alanine, L-valine, and L-proline [30] were elevated significantly in FANCC-high cells compared to the FANCC-low cells. Those metabolites are the signatures associated with aging inhibitory cellular processes, and FANCC thus can be directly implicated to also play crucial roles in the protection of humans from aging. More importantly, we also found that the levels of metabolites [the inosine [31] l-leucine, l-isoleucine, l-phenylalanine [32], hypoxanthine [33], and L-arginine [34]] were substantially changed in FANCC-high cells, which were directly related to inflammatory disease periodontitis (Figure 4B). Interestingly the level of inosine is 20 times higher in the cells carrying a high level of FANCC expression. Furthermore, we found the metabolites leading to another metabolite-disorder diabetes-mellitus (DM) (fumaric acid, fatty acid, D-glucose, carbon monoxide, collagens, 2-oxoglutaric acid) were altered in FANCC-high cells (Figure 5 A). Among those metabolites, signature ones (fumaric acid and collagens [35,36] to inhibitory DM processes were elevated (colored) in FANCC-high cells. While those metabolites that were anticipated to promote DM [35,36], however, were decreased (uncolored). Together, these findings suggest that FANCC is involved in the regulation of various disorders from cancer, aging, to inflammation as well as to DM, consistent with DM-prone mice deficient in FANCC expression [18].

## DISCUSSION

A growing number of studies have used mass spectrometry as a toll for the biomarker discovery [37, 38], but these studies have been largely cross sectional, provided limited information regarding the relation of metabolomic (or proteomic) biomarkers to the future development of diseases, thus metabolomic studies, particularly relevant to specific gene functions, are very important. The FA pathway has emerged as a curtail signaling network involving at least 22 FA proteins and many others together in the maintenance of multiple aspects of cellularr processes, which protects humans from aging, cancer, bone marrow failure, and many developmental defects [8,6,39]. However, it is rarely studied as to the relationship between cell metabolism and FA signaling /any individual FA protein. In this study, we showed how FANCC affected cellular biological functions through systematically studying FANCC-driven metabolic features and reveled the metabolomics aspects of inflammation, aging, or/and diabetes are associated with cancer at the metabolic level (Figure 1-5). Beside Warburg effect [40], other factors that link cancer to the metabolic disorder, include the referential metabolism of specific amino acids, such as valine, isoleucine, and glutamine [15,19,41]. In our experiment, we found that ten metabolites inosine, hypoxanthine, pyrophosphate, L-asparagine, L-phenylalanine, acetyl-L-carnitine, beta-alanine, glutarylcarnitine, niacinamide and creatine were significantly upregulated in FANCC-high cells (Figure 5), supporting the understanding that there is a reversible link between cancer and the metabolic disorder.

Previous reports suggest that inspection of the functional characteristics of a large set of interacting proteins demonstrates that they represent several general categories of cellular functions including involvement in transcription, cell signaling, transporter functions and oxidative metabolism. FANCC regulates oxidative metabolism as well as cell signaling functions by regulating cytoplasmic localization and interacting proteins associated with intracellular transport [42]. One large group of potential FA protein interactors are transcription regulators and proteins associated with transcription regulation was previously reported [43]. In our experiments, we found that FANCC proteins regulates the various metabolic signaling pathways that regulate cancer. Among all metabolites, L-ornithine, L-cysteine, L-asparagine, L-serine and docosahexaenoic acid were found to directly regulate the cancer (Figure 4C). This observation is consistent with the understanding of the multiple functions [42,43] that FA proteins perform.

A looming challenge in cancer metabolism is to begin to understand metabolic heterogeneity within intact tumors. It is already clear from mouse model that both the driver and tissue of origin influence metabolism when the tumor is considered as a single compartment [44]. It is much less known about heterogeneity within each individual tumor as to the regional differences in nutrient availability, localized effects of stromal and inflammatory cells, and the cell-autonomous effects regulated by clonal expression of mutants and all alter metabolic preferences and flexibility. Here we found that metabolites related to inflammation was expressed such as L-arginine, Hypoxanthine, L-phenylalanine, L-lysine, L-leucine, L-isoleucine and inosine (Figure 5A). Inosine was the most expressed metabolite in FANCC-high cells, these metabolites could trigger the environment that induces inflammatory responses, activates metabolite pathways and leads to metabolic disorders, inflammation and ultimately cancer. These observations and suggestions are consistent with the indications from the reported studies on cancer cells secreting lactates that acidify the microenvironment, trigger an inflammatory response and ultimately promote tumor progression [45], together strengthening our understanding about metabolic heterogeneity during cancer progression.

Interestingly, DM is not associated with patients with FA; however, mice with FANCC knockout showed DM-prone [18]. This suggested that DM may be attributed more to the deficient FANCC, instead of a defective FA signaling pathway. We thus profiled metabolites in the MDA-MB-321 breast cancer cells with and without ectopically- expressed FANCC (Figure 1). These cells harbor an endogenous defect in the FA signaling pathway owing to the absence of chromosome 15 (ATCC), where a key FA gene (FANCI) is located (Figure 1A and Figure S1A, B), and thus, can provide a better system to study the functions of an individual FA protein. Functional detection of FANCC-high or low cells showed that FANCC can suppress tumorigenesis as evidenced by the decreased cell proliferation and reduced colony formation in soft agar and decreased capacity in cell migration (Figure 1C, D, Figure S1C, D). Therefore, this study not only helps our understanding of how FANCC protects us from DM but also provides in-depth understanding of mitigated tumorigenecity at the metabolic level. Via GC−TOFMS and UPLC−MS/MS, annotated metabolomes of FANCC-high or low cells showed a clear separation (Figure 2). Detailed analyses of these specific differences were clearly indicated that FANCC may play roles in inflammation, diabetes, aging as well as cancer (Figures 2-5). As reported by Hotamislingil’s group, there was a link between inflammation and metabolic disorders. Many of the same metabolites are involved in obesity and diabetes, importantly few of the classic features for inflammation also had been observed in their metabolic studies on diabetes [46], and the subject link was indeed observed in our studies. More importantly, inflammation, diabetes along with aging can be suggested to be “a low grade” of cancer. This is because when FANCC is elevated, the tumorigenecity is mitigated in coordinated with the changed signature metabolites for aging, inflammation or diabetes. This condition may be fundamentally triggered by FANCC-driven metabolic alterations that engage a similar set of molecules and signaling pathways. Such as, we found that carbon monoxide, collagens, fatty acid, fumaric acid around to be involved in diabetes mellitus whereas inosine, L-arginine, hypoxanthine, l-phenylalanine, L-isoleucine, L-leucine, and L-lysine are involved in inflammatory disease periodontitis. From these FANCC-induced metabolic changes, we believe there is a principal link among cancer, aging, inflammation, and DM (Figure 5B), that mediates many metabolic disorders that can serve as “a low-grade status” of a specific disease, e.g., cancer.

## MATERIALS AND METHODS

### Cell culture and chemicals

Human breast cancer cell line MDA-MB-231 was obtained from the America Type Culture Collection (ATCC, Manassas, VA). The cell lines were prepared as previously described [47]. For the experiments, cells were trypsinized, and one million cells were counted and washed with 1X PBS; cell pellets were frozen at −80 °C. A total of three replicates were prepared for each cell types. Ethanol, pyridine, methoxyamine hydrochloride, C8−C30 fatty acid methyl esters (FAMEs), and ammonium acetate were purchased from Sigma-Aldrich (St. Louis, MO). Liquid chromatography−mass spectrometry (LC−MS) Optima-grade methanol and acetonitrile, formic acid, N-methyl-N-trimethyl –silyl-tri-fluoro-acetamide (MSTFA) with 1% TMCS, and hexane were obtained from Fisher Sci. (Fair Lawn, NJ). The ultrapure water was produced by Millipore Advantage A10 system with an LC−MS Polisher filter (Billerica, MA).

### Cloning, transfection and Western blotting

For cloning a full-length human FANCC, PCR amplification used primers (Forward: ATGGCTCAAGATTCAGTA, Reverse: CTAGACTTGAGTTCGCA). pcDNA3.1/NT-GFP-TOPO TA cloning and Expression (Kit from Invitrogen) and the building up of stable cell lines were carried out as shown previously [48]. For western blotting, protein samples were prepared from FANCC low or high group’s cells via using 1X SDS sample buffer, and separated in 8-10% SDS-PAGE and then transferred to the nitrocellulose membrane (Bio-Rad). Membranes were blocked with 5% non-fat skim milk in 1XPBS with tween-20 then incubated with FANCC and FANCI antibody diluted 1:1000 in 1XPBST. Horseradish peroxidase-conjugated rabbit or mouse antibodies were used as secondary antibody (1:5000-1:10000 dilution) for 1 hour at room temperature.

### Trans-well and soft -Agar assays

Trans-well assay was carried out using Matrigel Invasion Chamber, 24 well plates 8.0 Micron purchased from Coring. Briefly, 2X10^4^ cells were cultured in Matrigel well plates with two types of cells (Control and FANCC) respectively. Cells that were not migrated were swapped; while cells that migrated into the other side were fixed and stained with crystal violet. The images and numbers of migrated cells were used to indicate cell migration capacity. For soft Agar assay, 2X DMEM/F12 media was used to prepare 0.6% of base agar in 35mm dish and kept 20 minuses in 4ᴼC to make agar completely solidified, 2X10^3^ Cells were plated along with 2XDMEM/F12 in 0.4% of top ager. The dishes were incubated for 2 weeks at 37◦C and stained with crystal violet.

### Sample preparation for metabolic profiling

The cell line samples were prepared as previously described with modifications [49,50]. The appropriate weight of homogenizer beads and 50 μL of cold water were added to the cell-line samples for the first step of extraction. An aliquot of 270 μL of the mixture of ethanol and chloroform (3:1 = v/v) was added to the extracts for the second-step extraction. The sample extracts were centrifuged at 4 °C and 14500 rpm for 20 min. The supernatant was used for targeted metabolic profiling of lipids with Ultra-Performance Liquid-Chromatography tandem Mass Spectrometry (UPLC−MS/MS) and for untargeted metabolic profiling with Gas Chromatography -Time-of-Flight Mass Spectrometry (GC−TOFMS).

### Lipid profiling

Each aliquot of 20 μL of the supernatant was added to a 96-well Biocrates Kit plate (Biocrates Life Sciences, Austria) for lipid quantitation. After samples were dried under nitrogen, each 300 μL of extraction solvent (5 mM ammonium acetate in methanol) was added, and the kit plate was gently shaken at room temperature for 30 min. The sample extracts were filtered through the 0.45 μm membrane of the kit plate, and each aliquot of 20 μL of sample was diluted with 380 μL of methanol with 5 mM ammonium acetate for flow-injection analysis (FIA) of lipids.

Each 10 μL of the sample was directly injected into the mass spectrometer with elution solvent (methanol with 5 mM ammonium acetate) at a varied flow rate from 30 to 200 μL/min within 3 min.

### Untargeted metabolomics profiling with GC-TOFMS

Each aliquot of 250 μL above the supernatant was dried with a freeze-dryer. The dried samples were derivatized by methoxyamine hydrochloride in pyridine and subsequently by MSTFA. Retention indices of C8−C30 fatty acid methyl esters (FAMEs) were added for retention-time correction.

Each 1 μL sample was analyzed on an Agilent 7890A gas chromatography coupled to a Leco Pegasus time-off-light mass spectrometer (Leco Corp., St Joseph, MI) for global metabolite profiling. The analytes were introduced with a splitless mode to achieve maximum sensitivity and separated on an Rtx-5 MS capillary column (30 m × 0.25 mm I.D., 0.25 μm) (Restek, Bellefonte, PA). The column temperature was initially set to 80 °C for 2 min, increased to 300 °C for 12 min, and maintained at 300 °C for 5 min. The solvent delay was set to 4.4 min. The front inlet temperature, transferline temperature, and source temperature were set to 260 °C, 270 °C, and 220°C, respectively. The mass spectrometer was operated on a fullscan mode from 50 to 500 at an acquisition rate of 20 spectra/sec.

### Statistical data analysis

The raw LC−MS/MS data files were processed with TargetLynx Application Manager (Waters Corp., Milford, MA) to extract peak area and retention time of each metabolite. The raw GC−TOFMS data files were processed with Chroma TOF software (Leco Corp., St Joseph, MI) to extract peak signal and retention time for each metabolite. The detected metabolites were annotated with our internal standard library using an automated mass spectral data processing (AMSDP) software package [51]. IPA was applied to visualize the overall difference between FANCC-expressing high and low cells along with SIMCA-P 12.0.1 (Umetrics, Umeå, Sweden). Nonparametric statistical analysis (i.e., the Mann−Whitney U test) was used for searching the significantly different metabolites between the groups with a critical p-value of 0.05 and 0.01.

### Molecular pathway and network analysis in Ingenuity Pathway Analysis (IPA)

To systematically understand the metabolites in cells expressing a high level of FANCC compared to control cells expressing a basal level of FANCC, we uploaded the metabolite lists (with HMDB IDs) and the change folds of the differentially expressed metabolites onto an Ingenuity Pathway Analysis (IPA) server (http://3cr-apps.cc.hawaii.edu/IPA/). Canonical pathways and chemical−protein interaction networks were generated on the basis of the knowledge sorted in the Ingenuity Pathway Knowledge base. A ratio of the number of metabolites that map to the canonical pathway divided by the total number of molecules that map to the pathway was displayed. Fisher’s exact test was used to calculate the p-value to determine the probability that the association between the metabolites and the canonical pathway was explained by chance alone. The network score was based on the hypergeometric distribution and was calculated with the right-tailed Fisher’s exact test. The higher a score was, the more relevant the eligible submitted molecules were to the network.

## Supplementary Material

Supplementary File
