# Supplementary material for: Fanconi Anemia complementation group C protein in metabolic disorders
Source: Aging (Albany NY). 2018 Jun 21;10(6):1506–22. doi: 10.18632/aging.101487 (PMC6046246; doi:10.18632/aging.101487)
Supplement: Supplementary File [file aging-10-101487-s001.pdf]

SUPPLEMENTARY MATERIAL

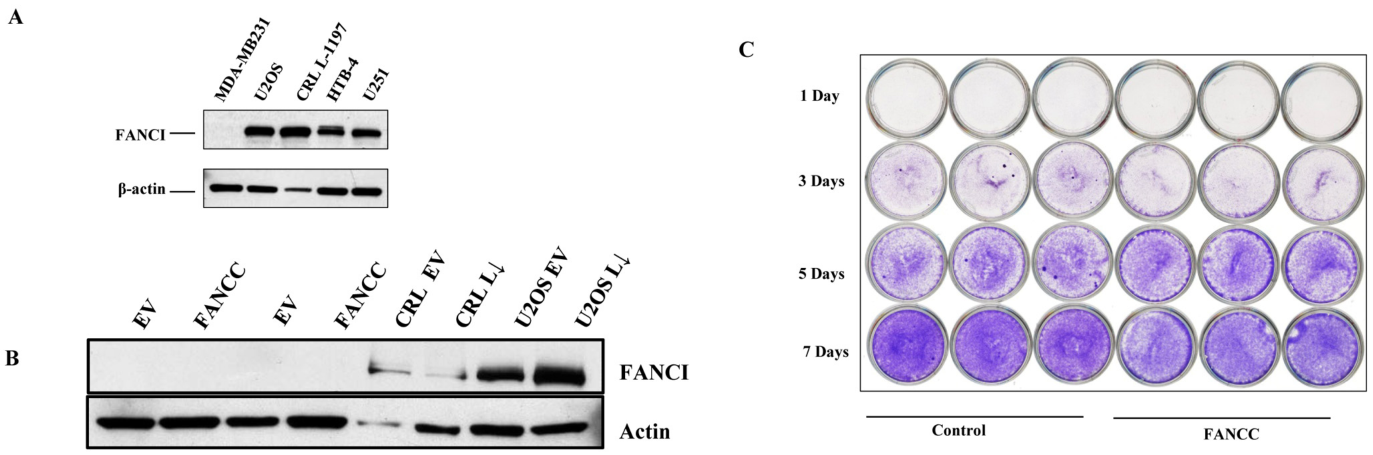

Supplementary Figure 1. (**A,B**) The FANCI expression is undetectable in MDA-MB231 cells. Additional western blotting showed that FANCI is not detectable in the parental cells (A) as well in derivatives (transfected with empty Vectors for control or FANCC cDNA containing plasmids) (**B**). (**C**) Cells containing FANCC cDNA grow slowly. Determination of the cell proliferation of FANCC-high versus low cells. A total of 2000 cells of FANCC-high or low cells were plated in triplicates and stained with crystal violet at the day 1, 3, 5 or 7. FANCC-high cells clearly have a lower density compared to the corresponding FANCC-low cells.

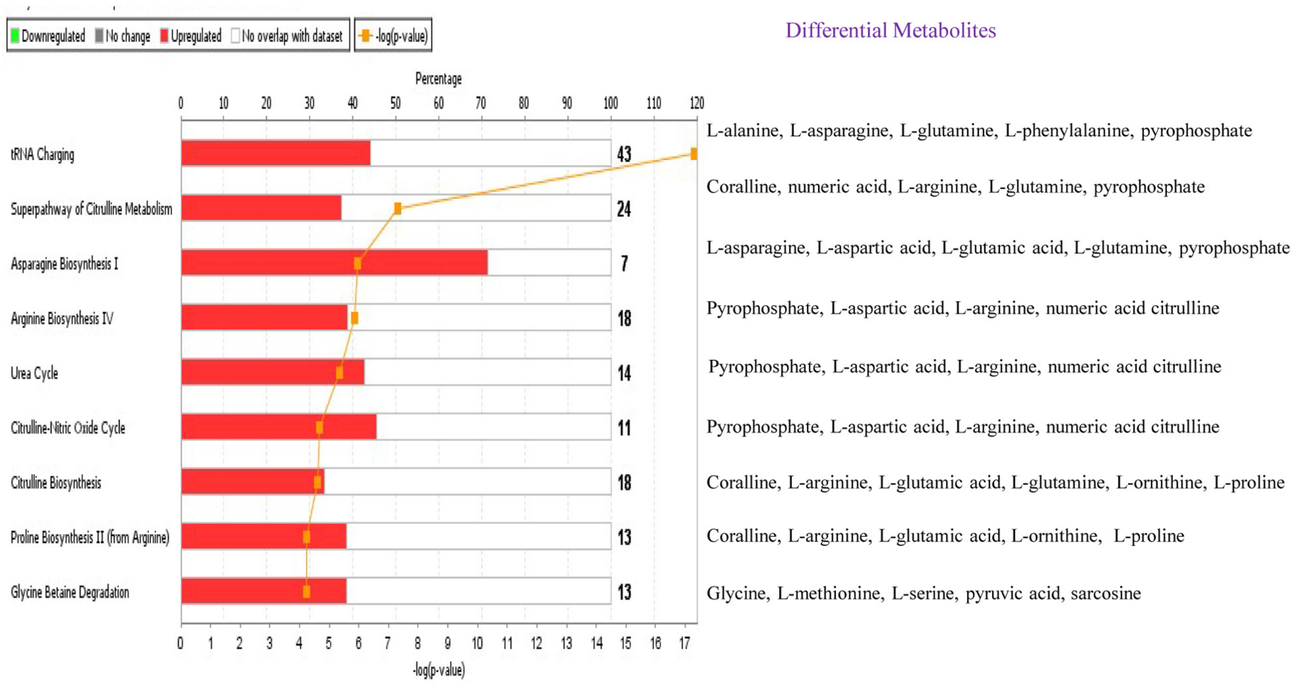

Supplementary Figure 2. Altered basic cellular processes in FANCC-high cells compared to FANCC-low cells. Analysis of molecular processes on the basis of metabolites present in FANCC-high or Low cells. Most significantly altered basic molecular rocesses and their metabolites revealed by IPA displayed. A total number of signature metabolites for each process were listed at the side.

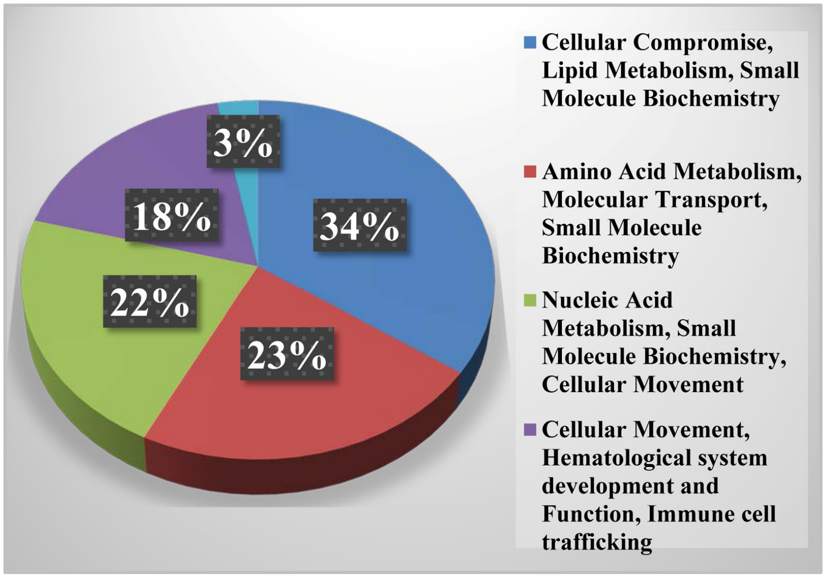

Supplementary Figure 3. Categories of altered 205 metabolites.

Supplementary Table 1. Summary of metabolomics analysis.

Canonical Pathway	p-Value	Overlap
tRNA Charging	5.1×10^{-18}	44.2% (19/43)
Superpathway of Citrulline Metabolism	4.9×10^{-08}	37.5% (9/24)
Arginine Biosynthesis IV	1.3×10^{-6}	71.4% (5/7)
Asparagine Biosynthesis	1.8×10^{-6}	38.9% (7/18)
Urea Cycle	4.33×10^{-6}	42.9% (6/14)
**Diseases and Bio Functions**	**p-Value**	**Metabolites**
Cancer	$4.39\times10^{-02} – 5.93\times10^{-14}$	30
Connective tissue Disorders	$3.67\times10^{-02} – 3.39\times10^{-07}$	15
Gastrointestinal Disease	$4.93\times10^{-02} – 5.93\times10^{-14}$	28
Inflammatory Disease	$3.67\times10^{-02} – 3.39\times10^{-07}$	7
Organismal injury and Abnormalities	$4.42\times10^{-02} – 5.9\times10^{-07}$	36
**Molecular and Cellular Functions**	**p-Value**	**Metabolites**
Cellular growth and Proliferation	$4.93\times10^{-02} – 5.93\times10^{-14}$	30
Amino Acid metabolism	$3.89\times10^{-02} – 2.43\times10^{-10}$	21
Molecular Transport	$4.93\times10^{-02} – 2.43\times10^{-10}$	33
Small Molecule Biochemistry	$4.93\times10^{-02} – 2.43\times10^{-10}$	36
Cell Cycle	$4.93\times10^{-02} – 2.43\times10^{-10}$	13
**Physiological System Development and Functions**	**p-Value**	**Metabolites**
Organism Development	$3.36\times10^{-02} – 8.20\times10^{-10}$	22
Hepatic system Development and Function	$3.67\times10^{-02} – 5.85\times10^{-11}$	11
Nervous System Development and Function	$4.93\times10^{-02} – 2.13\times10^{-05}$	15
Organismal Survival	$8.14\times10^{-04} – 8.14\times10^{-04}$	13
Tissue Morphology	$4.04\times10^{-02} – 7.57\times10^{-03}$	8

Toxicology Functions		
**Clinical Chemistry and Hematology**	**p-Value**	**Metabolites**
Increased Levels of albumin	$8.20\times10^{-10} - 2.80V10^{-10}$	8
Increase Levels of LDH	$1.92\times10^{-01} - 1.39\times10^{-01}$	3
Increase Levels of Alkaline Phosphatase	$1.32\times10^{-03} - 1.32\times10^{-03}$	2
Increase Levels of Creatinine	$1.32\times10^{-03} - 1.32\times10^{-03}$	2
**Cardiotoxicity**	**p-Value**	**Metabolites**
Cardiac Damage	$1.06\times10^{-01} - 1.06\times10^{-01}$	1
Cardiac Degeneration	$1.06\times10^{-01} - 1.06\times10^{-01}$	1
Bradycardia	$1.71\times10^{-01} - 1.71\times10^{-01}$	1
Congenital Heart Anomaly	$1.39\times10^{-01} - 1.39\times10^{-01}$	1
Cardiac Necrosis/Cell Death	$1.39\times10^{-01} - 1.39\times10^{-01}$	2
**Hepatotoxicity**	**p-Value**	**Metabolites**
Liver Necrosis/Cell Death	$7.34\times10^{-01} - 4.38\times10^{-03}$	6
Liver Steatosis	$2.31\times10^{-01} - 2.96\times10^{-04}$	7
Hepatocellular Carcinoma	$3.29\times10^{-06} - 3.29\times10^{-06}$	8
Liver Hyperplasia/Hyperproliferation	$3.29\times10^{-06} - 3.29\times10^{-06}$	8
Liver Inflammation/Hepatitis	$3.03\times10^{-01} - 3.67\times10^{-02}$	3
**Nephrotoxicity**	**p-Value**	**Metabolites**
Kidney Failure	$2.33\times10^{-01} - 3.67\times10^{-02}$	2
Renal Necrosis/Cell Death	$1.06\times10^{-01} - 3.67\times10^{-02}$	5
Glomerular Injury	$1.06\times10^{-01} - 1.06\times10^{-01}$	1
Nephrosis	$1.06\times10^{-01} - 1.06\times10^{-01}$	1
Renal Fibrosis	$1.06\times10^{-01} - 1.06\times10^{-01}$	1

Supplementary Table 2. Top analysis-ready molecules.

Experimental Fold Change up-regulated / down-regulated Molecules	
**Metabolites**	**Fold Change**
Inosine	⬆ 20.990
Hypoxanthine	⬆ 12.640
Pyrophosphate	⬆ 8.310
L-asparagine	⬆ 7.240
L-phenylalanine	⬆ 7.130
Acetyl-L-carnitine	⬆ 6.550
Beta-alanine	⬆ 6.300
Glutarylcarnitine	⬆ 6.110
Niacinamide	⬆ 6.030
Creatinine	⬆ 5.910
14:0/22:0 phosphatidylcholine	⬇ -8.400
22:1(13Z)/20:3(5Z,8Z,11Z) phosphatidylcholine	⬇ -5.880
18:0/24:1(15Z) phosphatidylcholine	⬇ -5.610
20:0/22:2(13Z,16Z) phosphatidylcholine	⬇ -5.590
20:0/20:2(11Z,14Z) phosphatidylcholine	⬇ -5.530
20:0/22:5(7Z,10Z,13Z,16Z,19Z) phosphatidylcholine	⬇ -5.380
16:0/24:1(15Z) phosphatidylcholine	⬇ -5.320
20:0/22:6(4Z,7Z,10Z,13Z,16Z,19Z) phosphatidylcholine	⬇ -4.910
18:3(9Z,12Z,15Z)/22:0 phosphatidylcholine	⬇ -4.700
dm18:0/18:1(9Z) phosphatidylcholine	⬇ -4.680

Supplementary Table 3. Top networks of the metabolites.

ID	Associated Network Functions	Score
1	Cellular Compromise, Lipid Metabolism, Small Molecule Biochemistry	35
2	Amino Acid Metabolism, Molecular Transport, Small Molecule Biochemistry	23
3	Nucleic Acid Metabolism, Small Molecule Biochemistry, Cellular Movement	22
4	Cellular Movement, Hematological system development and Function, Immune cell trafficking	18
5	Cellular Growth and Proliferation, Organism Development, Organismal Injury and Abnormalities	3
